# Interleukin-6 gene polymorphisms in Egyptian children with febrile seizures: a case–control study

**DOI:** 10.1186/s13052-016-0244-9

**Published:** 2016-03-09

**Authors:** Seham F. Azab, Mohamed A. Abdalhady, Alshaymaa Ali, Ezzat K. Amin, Dina T. Sarhan, Eman M. Elhindawy, Mohamed A. A. Almalky, Ahmed A. Elhewala, Mohamed M. A. Salam, Mustafa I. A. Hashem, Attia A. Soliman, Nagwa E. Akeel, Sawsan H. Abdellatif, Sanaa M. Ismail, Nahla A. Elsamad, Manal S. Arafat, Anwar A. Rass, Maha A. A. Basset

**Affiliations:** Faculty of Medicine, Zagazig University, 18 Omar Bin Elkhattab St, Al Qawmia, Zagazig City, AlSharqia Governorate Egypt; Mansoura Student Hospital, Mansoura, Egypt

**Keywords:** Febrile seizure, Gene polymorphisms, Cytokines, Interleukin-6

## Abstract

**Background:**

Febrile seizures are the most common form of childhood seizures. Among pro-inflammatory cytokines, interleukin-6 is the key acute-phase cytokine. To date, only a few studies concerned the association of interleukin-6 gene polymorphisms with febrile seizures.In this study, we aimed to investigate 3 cytokine single-nucleotide polymorphisms situated at positions –174 (G/C), –572 (G/C), and –597 (G/A) in the promoter region of the interleukin-6 gene for the first time in Egyptian children with febrile seizures.

**Methods:**

This was a case–control study included 100 patients with febrile seizure, and matched with age, gender, ethnicity 100 healthy control subjects.

Interleukin-6 –174 (G/C), −572 (G/C), and −597 (G/A) polymorphisms were genotyped by polymerase chain reaction-restriction fragment length polymorphism (*PCR-RFLP*), while the serum IL6 levels were measured by *ELISA* method.

**Results:**

Compared to the controls subjects, the frequency of the −174 GG and −597 GG IL6 genotypes were observed to be increased in children with febrile seizures (OR: 4.17; 95 % CI: 1.86–9.49; *P* <0.01 and OR: 1.96; 95 % CI: 1.06–3.63*;P* <0.05, respectively). We found a significant positive association between the −597 GG genotype and susceptibility to complex febrile seizures as did the G allele at the same position (OR: 4.2; 95 % CI: 1.4–13.3 for the GG genotype; *P* <0.01) and (OR: 2.89; 95 % CI: 1.1–7.7 for the G allele; *P* <0.05 respectively). Our data revealed no association between IL6- genotypes and serum IL6 levels in patients with febrile seizures (*P* > 0.05).

**Conclusion:**

In conclusion, our data brought a novel observation that the presence of a G allele or GG genotype at the −174 and the GG genotype at the −597 positions of the promoter region of the interleukin-6 gene constitute risk factors for developing febrile seizures in Egyptian children. Moreover, we observed a significant positive association between the IL6 –597 GG genotype and susceptibility to complex febrile seizures as did the G allele at the same position. However, we found no association between IL6- genotypes and serum IL6 levels in patients with febrile seizures.

## Background

Febrile seizures are the most common form of childhood seizures. It has been reported that one in every 25 children in the population will experience at least one febrile seizure during their childhood [1]. Febrile seizures are defined by the International League Against Epilepsy as “elevated or rapidly rising fever of short duration associated with uncomplicated seizure that does not predispose to epilepsy and is not accompanied by any neurologic abnormality, no previous neonatal seizures or a previous unprovoked seizure, and not meeting the criteria for other acute symptomatic seizures in children between 6 months and 5 years of age [2]. Febrile seizures were classified into three types: simple, complex, and febrile status epilepticus [3]. While cohort studies reported that prognosis of febrile seizures is good, epilepsy is observed in 5.4 % of these patients [4]. The main modulator of the condition is the seizure threshold, which varies strongly between individuals and is influenced by age, maturation, and genetic predisposition [5].

During infection, pro-inflammatory cytokines such as interleukin 1B (IL-1B), interleukin 6 (IL-6) and tumor necrosis factor-a (TNF-a) participate in inducing acute-phase reaction including fever [6]. Cytokines also play an important role in the protection of neurons against ischemia and cytotoxic damage in prolonged febrile seizures [6]. A dual role of IL-6 in seizures has been demonstrated in experimental models. An earlier study by Biber et al. [7] confirmed that stimulation of astrocytes and brain slices of cortex with IL-6 induced adenosine A1 receptor mRNA which is a powerful endogenous anti-convulsive substance. Fakuda et al. [8] reported that interleukin-6 plays an anti-convulsive role in experimental hyperthermia-induced seizures which might suggest similar properties of this cytokine in children with febrile seizures. On the other hand, intranasal administration of IL-6 exacerbated the severity of seizures induced by pentylenetetrazole on models of FS, supporting a pro-convulsant effect [9].

Complex interaction between immune-inflammatory process, cytokines activation, and genetic factors is involved in febrile seizures pathogenesis [10]. Recent studies suggested that cytokine genes single nucleotide polymorphisms is, at least in part, an aetiopathogenetic factor in the manifestation of febrile seizures, particularly in sporadic cases [11–14]. To date, only a few studies concerned the association of interleukin-6 gene polymorphisms with febrile seizures and the susceptibility to febrile seizures.

On the basis of these considerations, we designed this study to investigate 3 cytokine single-nucleotide polymorphisms situated at positions −174 (G/C), –572 (G/C), and –597 (G/A) in the promoter region of the interleukin-6 gene for the first time in Egyptian children with febrile seizures.

## Methods

This was a prospective case–control study performed in Zagazig University Children Hospital, and outpatient clinics in the same hospital from October 2013 to August 2015.

One hundred children; who had febrile seizures as diagnosed in the Department of Pediatrics in the same hospital, were enrolled in this study. The age of the patients ranged from 6 months to 6 years (median, 38 months). Diagnosis of febrile seizures followed the criteria established in the 1989 International Classification of Epileptic Syndromes [15]. Febrile seizures were defined as events in infancy or childhood that usually occur between 6 months and 5 years of age and are associated with a fever, but without evidence of intracranial infection or a defined cause for the seizure. Simple febrile seizures are generalized in onset, last less than 15 min, and do not occur more than once in 24 h. Complex seizures last longer, have focal symptoms, and can recur within 24 h. The electroencephalogram (EEG) was normal for all patients or showed mild nonspecific abnormalities.

### Exclusion criteria

Patients with febrile seizures beginning at the age of 6 years or later, afebrile seizures, evidence of intracranial infection or epileptiform EEG traits.

One hundred healthy children, of comparable age and gender; who attended Pediatric Department for preoperative evaluation for elective surgery, without a history of febrile seizures were enrolled. Patients and controls belonged to the same ethnic group: African Caucasian. All patients and controls included were subjected to proper history taking, thorough clinical and detailed neurological examination.

### Blood sampling

Blood was obtained from patients within 30 min of the time of seizure, and divided into 2 portions: 2 ml of whole blood was collected into tubes containing EDTA, for genomic DNA extraction. Serum were separated immediately from remaining part of the samples and stored at _20 _C till the time of use. Control samples were collected and similarly analyzed.

### Genomic DNA extraction

Genomic DNA was extracted from EDTA whole blood sample using a spin column method according to the protocol (QIAamp Blood Kit; Qiagen GmbH, Hilden, Germany). DNA was stored at 20 _C until analysis.

### IL6- Genotyping

All subjects were genotyped for IL6- polymorphism by polymerase chain reaction–restriction fragment length polymorphism (PCR-RFLP) as described before [13]. However, the results in some cases were not promising; and the experts did not accept it; therefore, few cases were excluded.

### Measurement of serum interleukin 6 (IL6) levels

The concentrations of IL6 in serum were estimated using a double antibody sandwich ELISA (kit provided by Biosource EuropeS.A., Belgium) according to the manufacturer’s instructions by using standard curve.

### Statistical analysis

IL6 genotype and allele frequencies in patients and controls were tested for Hardy–Weinberg equilibrium and any deviation between the observed and expected frequencies was tested for significance using chi-square and Fisher exact tests. In addition, the odds ratios (ORs) and 95 % confidence intervals (CIs) were calculated as a measure of the association between interleukin-6 (−174, −572, and −597) SNP and febrile seizures. The Student *t* test and analysis of variance were used to compare numeric variables within groups, depending on the distribution of the data. *P* value < 0.05 was considered to be statistically significant. All data were analyzed using the Epi Info statistical software (version 6.2, World Health Organization, Geneva, Switzerland).

### Ethics

Informed parental consent was obtained to be eligible for enrollment into the study. The study was done according to the rules of the Local Ethics Committee of Faculty of Medicine, Zagazig University, Egypt. Our institutional review committee of ethical research approved the study.

## Results

Our study included 100 patients with FS (63 patients with Simple FS and 37 patients with Complex FS, their age ranged from 6 to 72 months (median 38 months), 51 males and 49 females) and 100 healthy control subjects whose characteristics are listed in Table 1. The control group were age and gender matched to children with FS. The mean age at the onset of febrile seizures was 18 months (range, 6–43months). Within the group with complex febrile seizures, 23 patients had experienced two or more seizures, five patients had experienced focal seizures, and nine patients had experienced prolonged seizures lasting longer than 20 min. Thirty-one patients had a family history of febrile seizures, and 15 patients had a family history of epilepsy. As expected, patients with FS had increased serum levels of IL6 compared to the control group (mean, 32.7 pg/ml versus 10.5 pg/ml; *P* < 0.01, respectively); (Table 1).

**Table 1:** Baseline clinical and laboratory data of the studied patients and control groups

	Patients	Controls	*P*
	*n* = 100	*n* = 100	
*Age, months*	38(6–72)	35(6–70)	>0.05^a^
*Gender (M/F)*	51/49	53/47	>0.05^b^
*Type of Febrile seizure :*			
*Simple*	63	-	-
*Complex*	37	-	-
*Age at onset of Febrile seizure (months)*	18(6–43)	-	-
*Family history :*			
*Febrile convulsions*	31	-	-
*Epilepsy*	15	-	-
*Serum IL6 pg/ml*	32.7 ± 5.9	10.5 ± 1.7	<0.01^c^

*Abbreviations*: *IL6* interleukin 6

Data are presented as *median (Range) or mean ± SD*

*P* value < 0.05 indicates a significant difference

^a^ *Mann–Whitney U* test

^b^Chi-square test

^c^ *Student t-test*

Compared to the controls subjects, the frequency of the −174 GG and −597 GG IL6 genotypes were observed to be increased in children with febrile seizures (OR: 4.17; 95 % CI: 1.86–9.49; *P* <0.01 and OR: 1.96; 95 % CI: 1.06–3.63*;P* <0.05, respectively); Table 2.

**Table 2:** Distribution of IL-6 genotypes in patients with febrile seizures (FS) and the control group

Gene Polymorphism	Genotype	Patients n(%)	Controls n(%)	*OR*(*95 % CI*)	*P*
IL-6 (−174)	Sample size	100	100		
	GG	34 (34)	11(11)	4.17(1.86–9.49)	<0.01
	GC	57(57)	73(73)		
	CC	9(9)	16 (16)		
IL-6 (−572)	Sample size	98	100		
	GG	69(70.4)	65(65)		>0.05
	GC	25(25.5)	33(33)		
	CC	4(4.1)	2(2)		
IL-6 (−597)	Sample size	95	100		
	GG	62(65.3)	49(49)	1.96(1.06–3.63)	<0.05
	GA	31(32.6)	48(48)		
	AA	2(2.1)	3(3)		
*Serum IL6 pg/ml*		32.7 ± 5.9	10.5 ± 1.7		<0.01^a^

*Abbreviations*: *IL6* interleukin 6, *FS* febrile seizures, *OR* odds ratio, *CI* 95 % confidence interval

Values in parentheses are percentages or data are presented as mean ± SD

*P* value < 0.05 indicates a significant difference

Chi-square test

^a^ (*Student t-test*)

No significant association was evident between IL6 genotype and a positive family history of febrile seizures or epilepsy among studied patients; (*P* <0.05).

We found a significant increase in the frequency of the G allele (OR: 1.84; 95 % CI: 1.21–2.8; *P* <0.01) at the −174 position in IL-6 among the febrile seizure patients; where a concomitant significant decrease in the frequency of the C allele at the same position was observed compared to the control group (OR: 0.54; 95 % CI: 0.36–0.82; *P* <0.01); Table 3. On the other hand, no significant differences were found between the 2 groups in the allele frequencies for the −572 and −597 positions; Table 3.

**Table 3:** Distribution of IL-6 Alleles Frequency in patients with febrile seizures (FS) and the control group

Gene Polymorphism	Alleles	Patients n(%)	Controls n(%)	*OR*(*95 % CI*)	*P*
IL-6 (−174)	Sample size	100	100		
	G	125(63)	95(48)	1.84 (1.21–2.8)	<0.01
	C	75(37)	105(52)	0.54(0.36–0.82)	<0.01
IL-6 (−572)	Sample size	98	100		
	G	163(83)	163(81)		>0.05
	C	33(17)	37(19)		
IL-6 (−597)	Sample size	95	100		
	G	155(82)	146(73)		>0.05
	A	35(18)	54(27)		

*Abbreviations*: *IL6* interleukin 6, *FS* febrile seizures, *OR* odds ratio, *CI* 95 % confidence interval

*P* value < 0.05 indicates a significant difference

Chi-square test

Of note, when patients with simple or complex febrile seizures were evaluated, there was a significant positive association between the −597 GG genotype and susceptibility to complex febrile seizures as did the G allele at the same position (OR: 4.2; 95 % CI: 1.4–13.3 for the GG genotype; *P* <0.01) and (OR: 2.89; 95 % CI: 1.1–7.7 for the G allele; *P* <0.05 respectively); Table 4. Meanwhile, no significant differences were observed between the 2 groups genotype or allele frequencies for the–174 and −572 positions; Table 4. However, we could not find any significant association between IL-6 genotype or allele frequencies and prolonged seizure duration among subgroups of complex seizure patients (*P* > 0.05).

**Table 4:** Comparison of Genotype and Allele Frequency of IL-6 Polymorphisms between patients with simple febrile Seizure (SFS) and those with complex febrile seizures (CFS)

Gene Polymorphism	Genotype/Alleles	SFS Group n(%)	CFS Group n(%)	*P*
IL-6 (−174)	Sample size	63	37	
	GG	6(10)	3(8)	>0.05
	GC	36(57)	21(57)	
	CC	21(33)	13(35)	
Alleles	G	48(38)	27(36)	>0.05
	C	78(62)	47(64)	
IL-6 (−572)	Sample size	61	37	
	GG	44(72)	25(68)	>0.05
	GC	15(25)	10(27)	
	CC	2(3)	2(5)	
Alleles	G	103(84)	60(81)	>0.05
	C	19(16)	14(19)	
IL-6 (−597)	Sample size	59	36	
	GG	32(54)	30(83)	<0.01
	GA	26(44)	5(14)	
	AA	1(2)	1(3)	
Alleles	G	90(76)	65(90)	<0.05
	A	28(24)	7(10)	
*Serum IL6 pg/ml*		31.5 ± 6.9	33.7 ± 5.6	>0.05^a^

*Abbreviations*: *IL6* interleukin 6, *SFS* Simple febrile seizures, *CFS* Complex febrile seizures

*P* value < 0.05 indicates a significant difference. Chi-square test. ^a^ *Student t-test*

Patients with simple febrile seizures had serum IL6 levels similar to those with complex febrile seizures (*P* > 0.05); Table 4.

Our data revealed no significant association between IL6- genotypes or alleles and serum IL6 levels in patients with febrile seizures (*P* > 0.05); Table 5.

**Table 5:** Association of IL6 genotypes and alleles with serum IL-6 levels in febrile seizures patients

*Gene Polymorphism*	*Genotype/Alleles*	*Serum IL6 (pg/ml*)	*P*
IL-6 (−174)	GG	37.5 ± 6.9	>0.05^a^
	GC	35.3 ± 4.5	
	CC	33.5 ± 5.7	
Alleles			
	G	36.7 ± 3.8	>0.05^b^
	C	35.5 ± 6.4	
IL-6 (−572)	GG	33.7 ± 4.5	>0.05^a^
	GC	35.5 ± 5.7	
	CC	38.4 ± 3.9	
Alleles			
	G	34.2 ± 5.3	>0.05^b^
	C	39.5 ± 4.6	
IL-6 (−597)	GG	45.5 ± 9.7	>0.05^a^
	GA	43.3 ± 5.5	
	AA	47.5 ± 7.9	
Alleles			
	G	44.5 ± 5.3	>0.05 ^b^
	A	49.7 ± 8.5	

*Abbreviations*: *IL6* interleukin 6

Data are presented as mean ± SD

*P* value < 0.05 indicates a significant difference

^a^Calculated by *ANOVA* test

^b^ *Student t-test*

## Discussion

Pro-inflammatory cytokines as a key factor of host response to infection induct fever, leukocytosis, and acute phase protein synthesis [6]. Among these cytokines, interleukin-6 is a cell signaling molecule that has been associated with many diseases, including inflammatory, neurological, vascular, and malignant processes [16, 17]. Because of potential immune-modulatory effects of IL-6 and its importance as a major pro-inflammatory cytokine, IL-6 gene polymorphisms might affect individual susceptibility to febrile seizures.

In the present study, we found a significant positive associations between febrile seizure and the interleukin-6 G allele at the −174 position (OR: 1.84; *P* <0.01) and the −174 GG or −597 GG genotypes (OR: 4.17, and 1.96; *P* <0.01; respectively), thus revealing that patients were more susceptible to febrile seizure. Furthermore, we detected a significant negative association between febrile seizure and the C allele at the −174 position (OR: 0.54; *P* <0.01) indicating that this association could represent a protective effect against febrile seizures. These results were concordant with those of Nur et al. [13] who reported that the presence of the G allele or the GG genotype at −174 and the GG genotype at −572 positions of the interleukin-6 promoter regions constituted risk factors for developing febrile seizures in Turkish children. These findings support the hypothesis that a positive genotype predisposes individual to febrile seizure. However, we did not find a significant association between febrile seizure and the genotype or allele frequencies for the −572 position of the IL-6 promoter regions as they did.

On the contrary, Shahrokhi et al. [14] studied IL6 gene (−174 and +565) SNPs on genomic DNAs of 90 Iranian children with febrile seizures, compared to 139 healthy subjects. They reported that the presence of the G allele or the GG genotype at +565 position reduced risk of FS, while the A allele at +565 position of the promoter regions was a constituted risk factor for developing FS. However, the previous study on Taiwanese population by Chou et al. [18] failed to detect any association between interleukin-6 promoter gene polymorphism and febrile seizures. The authors also stated that no significant difference was observed in the distribution of allele frequencies of the IL-6 promoter, IL-1b promoter, IL-1b exon 5, IL-8, IL-10, or the tumor necrosis factor (TNF − α), gene polymorphisms.

Discrepancies between previously published studies and our study might be explained by the differences in age; study design or geographic/ethnicity, or by gene-gene or gene-environmental interactions. The genetic predisposition to febrile seizures could be polygenic, with many variants in multiple gene loci, playing an important role.

Of note, our data confirms a significant positive association between the IL6 –597 GG genotype and susceptibility to complex febrile seizures as did the G allele at the same position (OR: 4.2 and 2.89, respectively). However, no significant relationship was evident between IL-6 genotype or allele frequencies and prolonged seizure duration among subgroups of complex seizure patients. Similar to our results, Nur et al. [13] found a significantly higher frequency of the GG homozygous variant at position −597 of the IL6 gene in complex cases compared to patients with simple febrile seizures.

A positive family history of febrile seizure is the most meaningful risk factor, and the more relatives affected, the greater the risk is [19]. On the basis of family studies, the febrile seizure susceptibility trait is inherited in an autosomal dominant manner with reduced penetrance in large families. Linkage analysis revealed seven putative febrile seizure loci, chromosomes 8q, 19p, 2q, 5q, 6q, 18p, and 3p, which are at least partly associated with epilepsy or afebrile seizures [20]. By contrast, the inheritance appears to be polygenic or multifactorial in small families or sporadic cases. Surprisingly, we did not observe any significant association of IL6 gene polymorphism with a positive family history of febrile seizures among the studied patients (*P > 0.05*), which was contrary to our expectations and to previous findings of recent reports [13, 14] that the GG genotype at −174 was observed to be higher in familial cases which comprised a risk factor for febrile seizures.

It may be assumed that environmental factors such as exposure to recurrent viral infections; especially in our developing countries and host defense response related to cytokine gene polymorphism may be the cause of sporadic cases of febrile seizures and can play a significant role in their manifestation. Certainly, the small number of studies concerning cytokine gene polymorphisms, particularly in Caucasian population, makes it difficult to express explicitly the hypotheses or concepts.

IL6 is a pleiotropic cytokine secreted by a variety of cells such as T-lymphocytes, macrophages, endothelial cells and epithelial cells. The systemic concentration of IL6 is mainly regulated at the level of expression, as IL6 is rapidly cleared from the plasma with a short plasma half-life of 20–60 min [21].

As expected, we found that mean systemic IL6 levels in patients with febrile seizure was markedly elevated compared to the control group. However, patients with complex febrile seizure had serum IL6 levels similar to those with simple febrile seizure. Many other studies [22–24] reported the same findings, together with our results, may support the pro-convulsant action of IL-6 in febrile seizures. Virta et al. [22] found that the plasma interleukin-6 levels and interleukin-1 receptor antagonist were significantly higher in patients with febrile seizures, compared with febrile control subjects and CSF IL-6 levels were detectable in all studied patients with FS.

Lehtimäki et al. [23] explained that increased serum levels of cytokines originate mainly from the endothelial cells of the brain vessels and partially from the CSF compartment. The authors added that increased brain pro-inflammatory cytokines decrease the threshold for individual seizures; supporting the suggestion that neuro-inflammation may contribute to epileptogenesis in the developing brain [22–24]. On the other hand, Güven et al. [25] studied serum interleukin 6 levels, adiponectin and leptin as adipocytokines in children with febrile seizures compared with febrile control children. This research concluded that elevated levels of these adipocytokines as acute phase reactants in FS and FC groups did not contribute to the development of FS.

Interestingly, we observed that there was a lack of association between IL6- genotypes or alleles and serum IL6 levels in our patients with febrile seizures (*P* > 0.05). Against our results, Terry et al. [26] reported that the IL6-174G/C polymorphism affects transcription by altering the serum level of IL6, with the C allele associated with lower plasma IL6 concentrations. Endler et al. [27] added that other promoter polymorphisms within the IL6 gene, such as the IL6-572G/C polymorphism or complex haplotypes of other polymorphisms had been discussed as influencing IL6 plasma levels during the period of febrile disease.

However, more studies on cytokine profiles in the serum of child patients with febrile seizures or epilepsy are needed for a definite conclusion.

To the best of our knowledge, we demonstrated for the first time the association between certain genotype, and allele frequencies in IL-6 gene with febrile seizure in the Egyptian population. However, the small sample size was one of our limitations in this study; we suggest that multicenter approaches may be necessary to attain larger sample size. CSF IL-6 and interictal cytokine levels were not measured in our patients with febrile seizures which were other limitations in our study.

## Conclusion

In conclusion, our data brought a novel observation that the presence of a G allele or GG genotype at the −174 and the GG genotype at the −597 positions of the promoter region of the interleukin-6 gene constitute risk factors for developing febrile seizures in Egyptian children. Moreover, we observed a significant positive association between the IL6 –597 GG genotype and susceptibility to complex febrile seizures as did the G allele at the same position. However, we found no association between IL6- genotypes and serum IL6 levels in patients with febrile seizures.

Finally, further studies and more genetic information on ethnicities from different parts of the world will provide an additional understanding of the possible role of cytokine gene polymorphisms in the susceptibility to febrile seizure and progression to epilepsy.

## Abbreviations

IL-6: interleukin-6
IL-1: interleukin-1
IL-1Ra: interleukin-1 receptor antagonist
SNPs: single nucleotide polymorphisms
FS: febrile seizure
EEG: Electroencephalography
ELISA: enzyme-linked immunosorbent assay
PCR: polymerase chain reaction
RFLP: restriction fragment length polymorphism
OR: odds ratio
CI: confidence interval
TNFa: tumor necrosis factor- a
